# Conference Reports: DATA ADMINISTRATION MANAGEMENT ASSOCIATION SYMPOSIUM Gaithersburg, MD May 7–8, 1990

**DOI:** 10.6028/jres.095.047

**Published:** 1990

**Authors:** Judith Newton

**Affiliations:** Information System Engineering, National Computer Systems Laboratory, National Institute of Standards and Technology, Gaithersburg, MD 20899

## 1. Introduction

Information and information technology will form an integral part of the way business competes in the 1990s. Organizations will have to deploy their information resources effectively to succeed, exploiting information to make better decisions and gain market advantage.

The increased focus on the management of data to deliver information requires progress in data administration; an emphasis on information as a corporate resource considered independently of process.

The Data Administration Management Association (DAMA) is the professional organization for Data Administrators. An international board oversees a loose federation of local chapters in the United States and Australia. The National Capital Region Chapter (NCR DAMA) has monthly meetings from September through April, as well as a Symposium in May.

NCR DAMA held its third annual Symposium at NIST on May 7–8, 1990. The theme this year was “Future Directions in Information Management.” Attended by 264 Federal and private industry data administrators, the Symposium was co-sponsored by NIST and DAMA International. The keynote speakers were Professor N. Venkatraman of MIT’s Sloan School of Management and Robert Curtice of A. D. Little, Inc.

This year, the Symposium was extended to 2 days to accommodate the expanded program of speakers, workshops, and tutorials. The first day included sessions addressing Management Issues and Practical Techniques. The second day was divided between four tutorials and eight workshops. The workshops, to be held on a continuing basis, are designed to be research tools which will develop useful products for the data administration community.

## 2. Speakers

The key topics covered by the speakers included:

1. Information technology and business transformation,
2. Selecting a data management tool strategy,
3. Developing decision support systems using a data architecture,
4. Institutionalizing data architecture,
5. Building and managing a data administration function, and
6. Data architecture: planning effectively.

## 3. Tutorials

The tutorials were designed to introduce participants to unfamiliar topics.

The first, James Kendrick’s “The New Management Challenge—Aligning Business and Technological Choices,” discussed the implementation of information management in the corporate environment on a global scale. Effecting information-based management is a political process requiring strategic alignment of business and technical choices.

Strategic alignment consists of four areas:

1. Technology exploitation—studies information technology’s potential to influence the organization’s policy and direction.
2. Technology leverage—considers how business strategies affect the formation of an information technology strategy and the existing organization.
3. Strategy implementation—focuses on traditional strategy and structure implementation with the additional requirement of information technology support.
4. Technology implementation—focuses on implementing information technology strategies and their effect on business structures.

Information provides corporate agility necessary to be successful in the 1990s. Strategic alignment allows organizations to adapt to changing business and technological circumstances.

Ronald Ross presented a tutorial on “Defining Business Rules on a Data-Driven Basis.” Business rules can be defined to information systems through an object-oriented paradigm. Two systems defined by this paradigm are:

1. Sensory system—any system that creates, changes, or deletes data, and in so doing applies rules reflecting desired or correct behavior; and
2. Delivery System—any system that delivers data to its users, through appropriate channels (e.g., screens, reports, etc.).

Rules which are applied to these systems in an object-oriented information environment include:

1. Data-centered rule—A system that updates a stored data type cannot deliver it, and vice versa;
2. Data-precedence rule—A system cannot be built until the sensory components for the data type(s) it retrieves have been built.
3. Data-bounded rule—A sensory system may manage data for one, and only one, object type.
4. Data-encapsulation rule—The sensory system for an object must enforce all rules constraining that object, with support for each rule being rendered one and only one time.

These rules can be applied to object aging. Object state changes over time can be recorded; for example, the events in the life of an order can be tracked as the order is processed in the business systems. A business rule such as “An order cannot be completed until a payment has been received” will be captured and enforced.

The next tutorial presented a discussion of the logical development of business rule capture. Barbara von Halle addressed “Business Rules and Database Design.”

Traditionally, databases have been designed to process data in the most efficient possible way without regard for the requirements of business rule enforcement. Shared data environments, however, demand attention both to data considered as a resource and to the exigencies of rules which impose restrictions on the use of data. They require a partnership between business users and information specialists.

The shared data environment consists of business facts (data structure) and a corresponding set of common business rules (data integrity). Both data elements and business rules about the data must have business custodians. In order to insure business rule compliance in the application databases, this issue must be addressed at the level of the logical data model.

The logical data model is an integrated picture of all data used by an organization. It provides a starting point for database design and/or integration of one set of data requirements into a shared data environment. By integrating business rules into the data model, a place is provided for documenting detailed business data independently from how it is stored or accessed.

Here is an example of a working methodology for designing relational databases to include business rules.

1. Identify tables.
2. Identify columns.
3. Adapt data structure to product environment: sequence of columns, space calculations, file allocations, database assignment, and database locking.
4. Design for business rules about entities.
5. Design for business rules about relationships.
6. Design for additional business rules about data elements.
7. Tune: scans, clustering, hashing, indexing, etc.

The fourth tutorial was “Balancing Data and Process Modeling,” given by Chris Gane. The object-oriented paradigm was taken as the reference point to integrate data flow and data structure models for information systems architecture. In this way, system developers can get the reusability benefits of object-oriented programming languages without the performance limitations.

An object-oriented modeling object consists of four components:

1. A data entity with associated relationships, attributes, and domains. This is identical with an entity in a data model. It has attributes (characteristics) and relationships with other objects.
2. A set of relevant object procedures. Some of these, such as standard data manipulation language operations, will be reusable. Complex logic may be associated with these operations.
3. A set of conditions (procedures that return only true or false). This is the place to store business rules for eventual use by generated code. These conditions may also serve to define events, which may trigger the execution of procedures.
4. Other relevant object constraints, for instance, state-transition and referential integrity constraints.

The benefits of this approach derive from the storage of all modeling information, procedures, conditions, and constraints of concern to any object in one central location. Reuse of procedures and constraints is expedited. It assists inheritance of procedures, conditions, and constraints as well as relationships and attributes. And it provides a common basis for the definition of objects and methods, independently of eventual implementation.

## 4. Workshops

Eight workshops were held, each oriented towards a different topic in data administration. These workshops will continue to meet, sponsored by DAMA chapters, and produce deliverables which will be of assistance to data administrators. Each will set its own timetable for meetings and development schedule for deliverables. The workshops will be centrally coordinated by a member of the International DAMA board.

The workshops and their leaders are:

1. Data Naming Standards—Arnold Barnett, Barnett Data Systems.
2. Defining the Mission and Function of Data Resource Management: Terms and Concepts—Mike Phillips, AIRS, Inc.
3. Data Oriented Deliverables Produced by a Systems Development Life Cycle—Greta Blash, Information Systems, Inc.
4. Effective Data Management in a Multiple DBMS Environment—Larry K. Dougherty, Signet Bank.
5. Essential Capabilities for CASE Tools from the Data Administration Point of View—Joe Oates, Life Cycle Technology.
6. Data Administration’s Role in Supporting End User Computing—Bruce Rosen, NIST.
7. Pros and Cons of Different Data Modeling Techniques—Sashi Sood, AMS, Inc.
8. Standards and Procedures for the Data Administration Function—Judith Newton, NIST.

## 5. Proceedings

The proceedings of this Symposium will be released as a NIST publication. The Proceedings of the First^1^ and Second^2^ Annual DAMA Symposia were published by NIST and copies are still available. The Fourth Annual Symposium will be held at NIST May 14–15, 1991.

## Footnotes

1 Newton, Judith J., and Spielman, Frankie E., editors, Data Administration: Management and Practice, Proceedings of the First DAMA Symposium, NIST Special Publication 500-159, National Institute of Standards and Technology, Gaithersburg, MD, October, 1988.

2 Newton, Judith J., and Spielman, Frankie E., editors, Data Administration: Standards and Techniques, Proceedings of the Second Annual DAMA Symposium, NISTIR 90-4292, National Institute of Standards and Technology, Gaithersburg, MD, April 1990.

